# Coupling of green and brown food webs and ecosystem stability

**DOI:** 10.1002/ece3.6586

**Published:** 2020-08-11

**Authors:** Akihiko Mougi

**Affiliations:** ^1^ Institute of Agricultural and Life Sciences Academic Assembly Shimane University Matsue Japan

**Keywords:** brown world, detritus, green world, mathematical model, resilience

## Abstract

Ecosystems comprise living organisms and organic matter or detritus. In earlier community ecology theories, ecosystem dynamics were normally understood in terms of aboveground, green‐world trophic interaction networks, or food webs. Recently, there has been growing interest in the role played in ecosystem dynamics by detritus in underground, brown‐world interactions. However, the role of decomposers in the consumption of detritus to produce nutrients in ecosystem dynamics remains unclear. Here, an ecosystem model of trophic food chains, detritus, decomposers, and decomposer predators demonstrated that decomposers play a totally different role than that previously predicted, with regard to their relationship between nutrient cycling and ecosystem stability. The high flux of nutrients due to efficient decomposition by decomposers increases ecosystem stability. However, moderate levels of ecosystem openness (with movement of materials) can either greatly increase or decrease ecosystem stability. Furthermore, the stability of an ecosystem peaks at intermediate openness because open systems are less stable than closed systems. These findings suggest that decomposers and the food‐web dynamics of brown‐world interactions are crucial for ecosystem stability, and that the properties of decomposition rate and openness are important in predicting changes in ecosystem stability in response to changes in decomposition efficiency driven by climate change.

## Graphical abstract

An ecosystem model was used to show that decomposers play a critical role in ecosystem stability and totally change earlier predictions on the relationship between nutrient cycling and ecosystem stability. Ecosystem stability can be greatly mitigated by efficient decomposers. Furthermore, an open system is less stable than a closed system. System stability peaks at an intermediate openness.

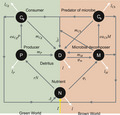

## 1 INTRODUCTION

Ecosystems are organized into aboveground green‐world pathways, which involve trophic interactions based on “green” primary producers, underground brown‐world pathways, which involve “brown” detritus‐based interactions, and the interaction between the two worlds. Community ecology is the study of biological interactions and their consequences as they relate to population dynamics and community stability and focuses mainly on green‐world pathways (May, 1972; McCann, 2000; Rosenzweig, 1971). Ecosystem ecology is the study of ecosystem functions, such as energy flow and material cycling, and focuses mainly on brown‐world pathways (Hobbie, 1992; Loreau, 2001; Wardle et al., 2004). Bridging the historical gap between these two disciplines remains a major challenge in ecological science (Hooper et al., 2005; Loreau et al., 2001; Rooney, McCann, Gellner, & Moore, 2006; Saint‐Béat et al., 2015; Wolkovich et al., 2014; Zou, Thébaul, Lacroix, & Barot, 2016).

A key step in the integration of the two disciplines would be to link ecosystem functions and the stability of population dynamics. Some pioneering work has been conducted in this area (DeAngelis, 1980; Loreau, 1994; Odum & Pinkerton, 1955; O'Neill, 1976). A general theory incorporating the flux of energy or biomass between species predicted that ecosystems are stabilized by an increase in flux rates (DeAngelis, 1980; Loreau, 1994). Although this prediction is supported by earlier modeling studies (Odum & Pinkerton, 1955; O'Neill, 1976), these earlier theories largely focused on aboveground primary productivity as the ecosystem function or did not explicitly consider brown‐world pathways. A recent study examined belowground dynamics (Miki, Ushio, Fukui, & Kondoh, 2010) but ignored aboveground food‐web dynamics. Although ecosystems include both aboveground and belowground dynamics, there is currently very little understanding about the role played by decomposers in nutrient cycling and food‐web dynamics and its effect on ecosystem stability.

Very few studies have developed ecosystem models to investigate the consequences of brown‐world pathways for the stability of population dynamics (Gounand et al., 2014; McCann, 2011), even though it has been predicted that primary producer‐based and detritus‐based food webs have qualitatively different impacts on food‐web stability (Moore et al., 2004; Moore, de Ruiter, & Hunt, 1993). McCann (2011) created an ecosystem model wherein detritus was seen to have a tendency to stabilize the entire ecosystem. More specifically, closed systems (that lack moving materials) become more stable with increasing flux rates, as predicted by earlier theories (DeAngelis, 1980; Loreau, 1994), whereas open systems (that possess moving materials) become less stable. Given that in nature a closed ecosystem is unrealistic, the presence of detritus is suggested to be a stabilizer of ecosystems, although efficient decomposition (high flux rate) can hinder this inherent stability. However, these predictions were based on a model that did not include the brown food web with decomposer dynamics, thus leaving open the question of how the presence of decomposers can affect ecosystem dynamics.

Here, an ecosystem model was developed to investigate the impact of microbial decomposers on ecosystem stability, which is defined as resilience via equilibrium recovery from a small perturbation (DeAngelis, 1980; McCann, 2011; Pimm & Lawton, 1977); however, future works will need to use multiple stability indices (Kéfi et al., 2019; Tilman, Reich, & Knops, 2006; Wang et al., 2017). The ecosystem comprised a green‐world pathway with nutrients, producers, and consumers and a brown‐world pathway with detritus, decomposers, and predators. Contrary to earlier theories, the results showed that ecosystem stability tends to be higher when the system has an intermediate degree of openness. However, in such stable systems with intermediate openness, high fluxes owing to efficient decomposition by decomposers can either greatly increase or decrease ecosystem stability, depending on the degree of openness. This suggests that an increased understanding of the properties of current ecosystems, such as decomposition rates and openness, is crucial for predicting changes in ecosystem stability in response to climate change and global warming.

## 2 MODEL

**FIGURE 1:**
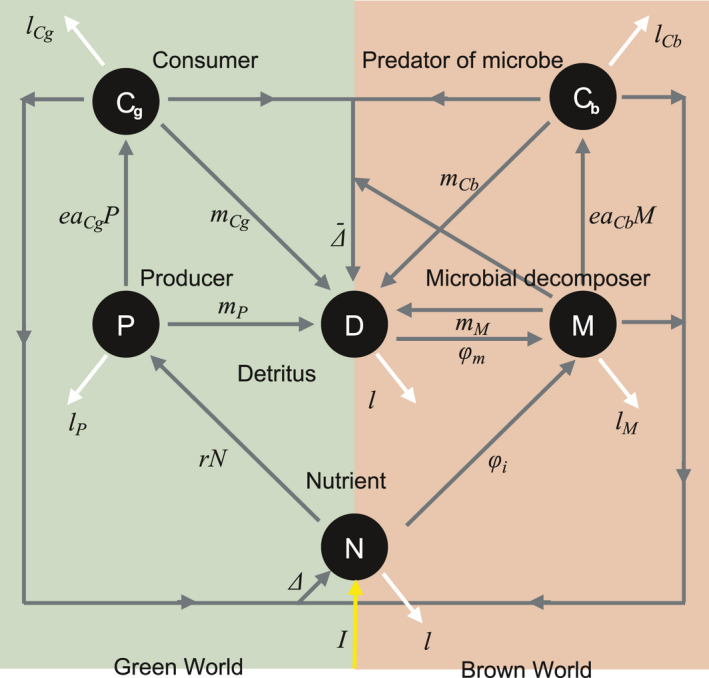
Schematic diagram of the ecosystem model. Circles represent compartments of nutrients (*N*), plants (*P*), consumers (*C_g_*), detritus (*D*), microbial decomposers (*M*), and predators (*C_b_*). The yellow arrow indicates nutrient input. The gray arrows indicate nutrient fluxes. The white arrows indicate nutrient loss

For the model, I considered an ecosystem wherein a classic food chain (green world) and a detritus–decomposer–predator chain (brown world) were coupled (Figure 1), assuming that decomposer population growth was limited either by carbon (C‐limited) or a mineral nutrient (N‐limited) (Daufresne, Lacroix, Benhaim, & Loreau, 2008; Daufresne & Loreau, 2001). Although food‐web structures can vary between ecosystems, here I assumed the simplest system with different functional groups. The robustness of the predictions was confirmed using a more complicated model, which included second consumers in both the green and brown worlds (Results). The ecosystem model was defined by the following ordinary differential equations:

(1a) $$\frac{\mathrm{dN}}{\mathrm{dt}}=I‐l_{N}N‐rNP‐\varphi_{i}+\Delta ,$$

(1b) $$\frac{\mathrm{dP}}{\mathrm{dt}}=rNP‐\left(l_{P}+m_{P}\right)P‐a_{\mathrm{Cg}}PC_{g},$$

(1c) $$\frac{dC_{g}}{\mathrm{dt}}=e_{\mathrm{Cg}}a_{\mathrm{Cg}}PC_{g}‐\left(l_{\mathrm{Cg}}+m_{\mathrm{Cg}}\right)C_{g},$$

(1d) $$\frac{\mathrm{dD}}{\mathrm{dt}}=\bar{\Delta }+m_{P}P+m_{\mathrm{Cg}}C_{g}+m_{M}M+m_{\mathrm{Cb}}C_{b}‐a_{M}DM‐l_{D}D,$$

(1e) $$\frac{\mathrm{dM}}{\mathrm{dt}}=\varphi_{m}‐\left(l_{M}+m_{M}\right)M‐a_{\mathrm{Cb}}MC_{b},$$

(1f) $$\frac{dC_{b}}{\mathrm{dt}}=e_{\mathrm{Cb}}a_{\mathrm{Cb}}MC_{b}‐\left(l_{\mathrm{Cb}}+m_{\mathrm{Cb}}\right)C_{b},$$

where *N*, *P*, *C_g_*, *D*, *M*, and *C_b_* are the nutrient pool size and the biomass of the producers, consumers, detritus, microbial decomposers, and predators of microbes, respectively. *I* is the nutrient input rate; *a_M_* is the decomposition rate; *e_M_* is the conversion efficiency of detritus into microbe production; *r* is the nutrient uptake rate of the producers; *a_Cg_* is the uptake rate of the producers by the consumers; *e_Cg_* is the conversion efficiency of the producers into consumer production; *a_Cb_* is the rate of uptake of microbes by predators; *e_Cb_* is the efficiency of conversion of microbes into predators; *l_i_* (*i* = *N*, *P*, *C_g_*, *D*, *M,* or *C_b_*) is the rate of nutrient loss from the system (a part of dead organisms can emigrate from the system, and the immigration can be negligible if the focus of the system is on a broad spatial scale because most of the area can be source and residual can be sink); and *m_i_* is the mortality rate of a species (*i* = *P*, *C_g_*, *D*, *M*, or *C_b_*).

Δ = *δ_Cg_*(1 − *e_Cg_*)*a_Cg_PC_g_* + *δ_Cb_*(1 − *e_Cb_*)*a_Cb_MC_b_* + *δ_M_*(1 − *e_M_*)*a_M_DM*, where *δ_i_* is a fraction of the nutrients released from all compartments added to the N pool; this represents direct nutrient cycling via excretory processes. $\bar{\Delta }$ = (1 − *δ_Cg_*)(1 − *e_Cg_*)*a_Cg_PC_g_* + (1 − *δ_Cb_*)(1 − *e_Cb_*)*a_Cb_MC_b_* + (1 − *δ_M_*)(1 − *e_M_*)*a_M_DM*, where 1 − *δ_i_* is a fraction of the nutrients released from all compartments added to the organic material pool as detritus; this represents indirect nutrient cycling by microbes via the mineralization of detritus, such as feces and dead organisms, before becoming available to producers.

The carbon/nutrient limitation of decomposers depends on differences between the C:N demand of decomposers and the C:N supplied by detritus (Bosatta & Berendse, 1984; Daufresne et al., 2008; Sterner & Elser, 2002). When there is a lower abundance of detritus and a lower C:N ratio as compared with decomposers, decomposers are C‐limited. In contrast, when there is an abundance of detritus and available carbon, decomposers are N‐limited (Daufresne et al., 2008; Daufresne & Loreau, 2001; Zou et al., 2016). Decomposer growth *φ_m_* and nutrient uptake by decomposers *φ_i_* are then assumed as follows:

(2a) $$\varphi_{m}=\text{Min}\left[e_{M}a_{M}DM\frac{\alpha }{\beta },e_{M}a_{M}DM+r_{M}NM\right],$$

(2b) $$\varphi_{i}=\text{Min}\left[e_{M}a_{M}DM\left(\frac{\alpha }{\beta }‐1\right),r_{M}NM\right],$$

where *α* and *β* represent the C:N ratio of detritus and decomposers, respectively (normally, *α* > *β* in systems wherein detritus is the substrate for the decomposer community (Ågren & Bosatta, 1996; Andersen, 1997)). Hereafter, *α*/*β* = *q* (>1)*. r_M_* is defined as the nutrient uptake rate of the decomposers. The left and right terms in each equation represent C‐ and N‐limitation, respectively. From Equations (2a) and (2b), switching between C‐ and N‐limited systems is determined by the following condition: if e_M_a_M_D^*^(*q* − 1) < r_M_N^*^, C‐limited, otherwise, N‐limited (Zou et al., 2016).

Efficiency was not considered in terms of the nutrient uptake of the producers or decomposers because their efficiencies were likely to be similar (McCann, 2011; Zou et al., 2016). Also, for simplicity, *e_i_* = *e*, *δ_i_* = *δ*, and *l_N_* = *l_D_* = *l* were assumed. By setting the right‐hand sides of Equations (1a), (1b), (1c), (1d), (1e), (1f) to zero, a nontrivial equilibrium is obtained (Appendix S1). Since this paper does not focus on a specific system, I assumed parameter values that allow species to coexist and can show general patterns of model behavior. In particular, I explored the effect of a key parameter in this paper, that is, decomposition rate (*a_M_* > 0). The range of this parameter is determined by the factors explained in the followings. First is the feasibility. The parameter range must include a range where all equilibrium values are positive. Second is the saturation of a pattern of the system. The system saturates to constant equilibrium values with increasing *a_M_*, under the feasible condition. Note that *P* ^*^ and *M* ^*^ are always positive constant without depending on *a_M_*, and *N* ^*^, $C_{g}^{*}$, and $C_{b}^{*}$ can converge to positive constant (Appendix S1). Importantly, *D* ^*^ approaches to zero with increasing *a_M_*. These factors indicate that the property of the system does not change at a sufficient large value of *a_M_*. For that reason, the upper limit of this parameter was determined.

Using numerical analysis, local stability was determined by examining an actual portion of the dominant eigenvalue. There were no unstable regions in this system (limit cycles could not occur); therefore, the coexistence equilibrium was assumed to be globally stable at all times. Hence, in the coexistence equilibrium, resilience (an index of ecosystem stability), defined as the capacity of a system to return to a stable equilibrium after encountering an acute disturbance, was calculated as the absolute value of the highest real part of the eigenvalues of the Jacobian matrix (Pimm & Lawton, 1977). To reveal the roles played by decomposers in ecosystem dynamics, I compared the stability of the systems with or without decomposers. To demonstrate the effect of decomposers alone, I assumed the flux into the nutrient pool of systems with or without decomposers to be equal. This is because productivity largely influencing stability can be different if this is not assumed (McCann, 2011). This comparison was done in systems that lacked consumers of decomposers, because it is not possible if it exists. In addition, I focused particularly on the effects of decomposition rate, which is a key parameter involved in the control of nutrient recycling within a system. By varying the degree of decomposition by microbes (*a_M_*) and the rates of nutrient and detritus loss (*l*), the effects of decomposition rate and the openness of the system on ecosystem stability were investigated, to reveal the roles played by decomposers in ecosystem dynamics.

## 3 RESULTS

**FIGURE 2:**
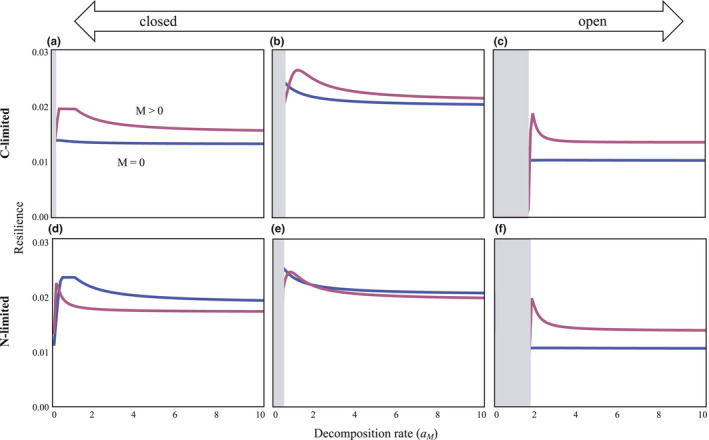
Relationships between total nutrient input and resilience in systems with or without decomposers. (a‐c) C‐limited. (d‐f) N‐limited. The systems do not include consumers of decomposers. Red and blue lines indicate systems with or without decomposers, respectively. In the gray regions, coexistence does not occur. Total nutrient input is controlled by keeping the total nutrient input into the nutrient pool of those systems equal. In both C‐ and N‐limited systems, $I^{\prime }+\delta \left(1‐e\right)a_{\mathrm{Cg}}P^{*\prime }C_{g}^{*\prime }+\delta \left(1‐e\right)a_{M}M^{*}D^{*\prime }=I^{\prime \prime }+\delta \left(1‐e\right)a_{\mathrm{Cg}}P^{*\prime \prime }C_{g}^{*\prime \prime }+a_{M}D^{*\prime \prime }$, where the apostrophe and double apostrophe indicate particular values of input rate and equilibria in systems with or without decomposers, respectively. The values of *I*″ are controlled in each value of *a_M_*. In (a), (b), (c), (d), (e), and (f), *l* = 0.01, 0.1, 0.2, 0.02, 0.1, and 0.2, respectively. Other parameter values are as follows: *I*′ = 1, *r* = 0.05, *e* = 0.25, *δ* = 0.5, *q* = 1.2, *a_Cg_* = 1, *r_M_* = 0.01, *m_P_* = 0.1, *m_Cg_* = 0.1, *m_M_* = 0.1, *l_P_* = 0.1, *l_Cg_* = 0.1, and *l_M_* = 0.1

The system had coexistence equilibrium in cases of both C‐ and N‐limitation (Appendix S1). First, consider systems without consumers of decomposers to reveal the role of decomposers in system stability (*C_b_* = 0). I analyzed the effects of decomposers on stability by comparing the systems with or without decomposers. The analysis showed that in a C‐limited system (Figure 2a‐c), decomposers could stabilize the system over a broad range of decomposition rates (*a_M_*) and openness (*l*). In an N‐limited system (Figure 2d‐f), however, stabilization due to decomposers was not likely to occur in nonopen systems. In both systems, decomposers can play a role in stabilizing the system, particularly if the system is open and the decomposition rate is not low (Figure 2). This tendency is almost qualitatively held even when consumption rate of producers changes (Figures S1 and S2). In addition, the presence of consumers of decomposers can play a stabilizing role in an ecosystem, particularly if the system is open and the consumption rate is high (Figures S3 and S4).

**FIGURE 3:**
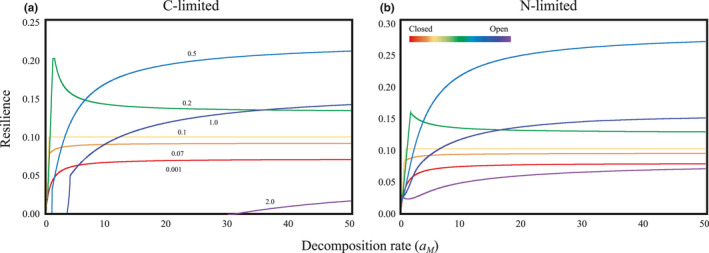
Relationships between the decomposition rate (*a_M_*) and resilience with varying degrees of ecosystem openness. (a) C‐limited. (b) N‐limited. Colors indicate different levels of ecosystem openness. Following an earlier study (O'Neill, 1976), the openness is controlled by the nutrient loss rates *l*. *I* = 2, *r* = 2, *e* = 0.25, *δ* = 0.5, *q* = 1.2, *a_Cg_* = 1, *a_Cb_* = 1, *r_M_* = 1, *m_P_* = 0.1, *m_Cg_* = 0.1, *m_M_* = 0.1, *m_Cb_* = 0.1, *l_P_* = 0.1, *l_Cg_* = 0.1, *l_M_* = 0.1, and *l_Cb_* = 0.1

Next, I examined the effects of decomposition rate on stability using the full model (Equations 1). Consider an extreme case where the system is almost closed (lower rate of *l*) (McCann, 2011). An increase in the decomposition rate owing to efficient decomposition tended to stabilize the system (Figure 3). This qualitative tendency remained essentially unchanged, even if the closed nature of the ecosystem was relaxed (Figure 3). However, openness had two intriguing effects on ecosystem stability. First, at an intermediate level of openness (e.g., *l* = 0.2; Figure 3), the stability peaked at an intermediate decomposition rate (the peak can be observed over 0.1 < *l* < 0.4). Stabilization was limited to lower decomposition rates, while a further increase in flux will reverse the stabilizing effect just after exceeding the threshold flux. Second, openness had a nonlinear effect on stability. Stability also peaked at intermediate openness (Figure 3). These patterns remained qualitatively unchanged, regardless of whether decomposer growth was C‐ or N‐limited (Figure 3) and over a wide parameter range (Figures S5‐S8). Even with the donor‐controlled function of decomposers (decomposers do not control the levels of detritus or nutrients), the major results remained true (Figure S9). In addition, second consumers in both green and brown worlds showed a qualitatively similar pattern in the relationship between stability and decomposition rate (Figure S10).

**FIGURE 4:**
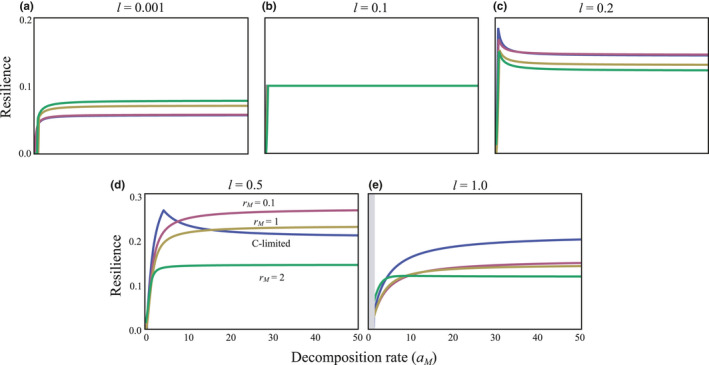
Relationships between the decomposition rate (*a_M_*) and resilience in C‐ and N‐limited systems. In (a)‐(e), the levels of openness are varied. Blue lines are C‐limited cases. The other lines are N‐limited cases, each of which has different values of *r_M_*. The parameters are as follows: *I* = 2, *r* = 1, *e* = 0.25, *δ* = 0.5, *q* = 1.2, *a_Cg_* = 1, *a_Cb_* = 1, *m_P_* = *m_Cg_* = *m_M_* = *m_Cb_* = 0.1, and *l_P_* = *l_Cg_* = *l_M_* = *l_Cb_* = 0.1

The decomposer growth type (C‐ or N‐limited) can affect stability. I compared the stability in each system, all other factors being equal. The influence of decomposer growth type on stability critically depended on the degree of openness. When openness was low or the system was closed, an N‐limited system exhibited higher stability than a C‐limited system, particularly where there was a high exploitation rate of nutrients by decomposers (*r_M_*) and high decomposition rates (Figure 4a). Conversely, when there was a high degree of openness, this tendency was reversed: A C‐limited system exhibited higher stability than an N‐limited system, particularly when there was a high exploitation rate of nutrients by decomposers (*r_M_*) and high decomposition rates (Figure 4c‐e).

## 4 DISCUSSION

The present theory predicts that (a) the presence of decomposers can play a stabilizing role in a system, particularly when the decomposition rate and degree of openness of the system are not low; (b) increased decomposition tends to stabilize the ecosystem; (c) increased decomposition produces a hump‐shaped effect on the stability of systems with an intermediate degree of openness; (d) ecosystems with a moderate degree of openness are highly stable; and (e) C‐limited systems tend to be more stable than N‐limited systems, particularly when a system is open and decomposers are efficient consumer. The present study suggests that ecosystems are stabilized by efficient decomposers, particularly when these systems have a moderate degree of openness.

The present study also suggests that the magnitude of the flux, or decomposition via the consumption of detritus by decomposers, on the whole tends to be positively correlated with stability. This prediction is known to be true for more simple systems. McCann (2011) demonstrated that in a system with detritus dynamics the same prediction holds if the system is one that is almost closed, while it can be completely reversed in open systems; an increase in the flux or decomposition rate tended to destabilize the system. However, the prediction based on the outputs of the present model, with more explicit brown‐world pathway participation (and with both detritus and decomposers), is, on the whole, a positive relationship between the magnitude of flux through decomposition and stability (at least, monotonical destabilization due to efficient decomposition was not observed). Interestingly, this prediction from a mechanistic model is quite similar to one from the simplest phenomenological model, which lacked both detritus and decomposers (DeAngelis, 1980; Loreau, 1994), while the unimodal pattern in flux (nutrient input rate) and stability was not observed in the earlier model. These differences suggest that the presence of decomposers can totally change the predictions made in relation to ecosystem dynamics.

Why do decomposers have a positive effect on stability? Regardless of the existence of microbial decomposers, detritus decreases as the decomposition rate increases, finally becoming depleted ($\lim_{a_{M}\to \infty }D^{*}=0$ is analytically shown in the Appendix S1). Thus, without microbes, the recovery of a system following a perturbation would be delayed, particularly when decomposition occurs relatively rapidly, because detritus does not play a role in circulating nutrients within a system. When microbes are present, however, even if detritus is less abundant, there may be a high abundance of efficient microbial decomposers ($\lim_{a_{M}\to \infty }M^{*}>0$ is analytically shown in Appendix S1), and the nutrients become tied up in biotic biomass. Hence, even if a perturbation occurs, microbes can play a key role in maintaining the smooth circulation of nutrients within the system. Therefore, the rate of microbial decomposition is a key parameter in stabilizing a system.

Why is stability highest at intermediate levels of decomposition rate and openness? This can be interpreted from two perspectives. The first is openness. Consider an extreme case where a system is much closed. In such a case, both detritus and nutrients are abundant, and these rich nutrients help consumers to grow; therefore, these consumers largely regulate plants and microbes. A scarcity of plants and microbes would delay the recovery of consumers and nutrients following a perturbation. Consider another extreme where a system is very open. In this case, in contrast to a closed system, both nutrients and detritus are very scarce. Hence, if a perturbation occurs, plants and microbes will be less likely to recover. This is one reason why an intermediate degree of openness exhibits high stability. The second point is the decomposition rate. Consider an extreme case where the decomposition rate is very low. In such a case, nutrients are scarce but detritus is abundant. Less abundant nutrients would result in the slower recovery of plants or the green world following a perturbation. Conversely, at the opposite extreme where the decomposition rate is very high, detritus is scarce but nutrients are abundant. A lower abundance of detritus would make the recovery of microbes or the brown world slower following a perturbation. This is one reason why an intermediate rate of decomposition exhibits high stability. This is likely to be a robust prediction, since some analysis of top predators has shown a qualitatively similar pattern (Figure S10), although further work is necessary.

These predictions have some important implications for ecosystem conservation. Ecosystems with a moderate degree of openness can either be stabilized or destabilized if the decomposition rate increases, depending on the current rate of decomposition. This finding suggests that further understanding of the current state of ecosystems is necessary to predict whether a change in decomposition efficiency driven by climate change, such as global warming, can stabilize an ecosystem (e.g., effect of temperature changes on decomposition efficiency (Davidson & Janssens, 2006)). Openness plays a key role in ecosystem maintenance. Earlier theories predicted that closed systems are less stable than more open systems (DeAngelis, 1980; Loreau, 1994; McCann, 2011). However, a totally different prediction was derived from the present model, with more open systems tending to be less stable than those systems that were almost closed. Furthermore, both almost closed and more open ecosystems are less stable than systems with an intermediate degree of openness. The contradiction between earlier theories and the present proposal suggests a crucial role for brown food webs in ecosystem dynamics.

Why does decomposer growth type affect ecosystem stability? The present results suggest that the effect of decomposer growth type on stability can differ depending on the level of system openness. More specifically, in closed systems, N‐limited tends to be more stable, particularly when the decomposition rate is not low. In contrast, this tendency is reversed in an open system: C‐limited is more stable. This can be explained as follows. When resources are unlikely to be depleted (closed system), nutrient use by decomposers in an N‐limited system can result in decomposers becoming more productive, promoting decomposition, and leading to efficient recycling and increased stability. However, when resources are likely to be depleted (open system), the result is totally different. In such a case, an N‐limited system plays a role in decreasing nutrients and promoting resource depletion, resulting in decreased stability. This mechanism suggests that stability can change markedly as a result of environmental changes which cause shifts in decomposer growth type or system openness, such as habitat destruction.

To the best of my knowledge, the most similar modeling study to the present one was carried out by Zou et al. (2016). Although a careful discussion is necessary because the system modeled was not the same as that in the present study, the following comparison may be possible. Zou and colleagues analyzed the links between the green and brown worlds. More specifically, they showed how some parameters in one world can influence the productivity in the other. In their model, productivity in the green world was a monotonical function of the rate of consumption of decomposers by predators. This suggested that the regulation of decomposition is closely related to plant productivity. A key point was that the productivity level is middle at an intermediate level of regulation of decomposers. In addition, high or low productivity in one world could lead to low or high productivity in the other world, respectively. This is because, for example, a high abundance of detritus implies stagnation of the brown world, resulting in a low level of nutrients, and vice versa. Hence, as well as the discussion on stability, mid‐level productivity could increase stability. This link between stability and ecosystem functioning will be the subject of future studies.

The present study highlights the potential importance of the decomposition rate and the degree of openness in ecosystem maintenance. However, empirical data linking ecosystem stability with decomposition rates are currently lacking. Consequently, additional empirical studies of the dynamics of ecosystems, along with both green and brown food webs, are warranted. A possible test of the present hypothesis would be to compare the stability of ecosystems that possess different degrees of openness (e.g., stream vs. lake (Essington & Carpenter, 2000)), under varying levels of decomposition efficiency (e.g., temperature (Davidson & Janssens, 2006)). In addition, further theoretical studies are necessary to confirm the robustness of this prediction. For instance, whether this prediction is applicable to nonequilibrium systems or meta‐ecosystems (Massol et al., 2011) remains an open and important question.

## CONFLICT OF INTEREST

None declared.

## AUTHOR CONTRIBUTIONS

**Akihiko Mougi:** Conceptualization (lead); formal analysis (lead); funding acquisition (lead); investigation (lead); methodology (lead); project administration (lead); supervision (lead); validation (lead); visualization (lead); writing – original draft (lead); writing – review & editing (lead).

## Supporting information

Supplementary Material

## Data Availability

This paper includes no data because it describes a mathematical model.
