# Supplementary material for: Coupling of green and brown food webs and ecosystem stability
Source: Ecol Evol. 2020 Aug 11;10(17):9192–9. doi: 10.1002/ece3.6586 (PMC7487232; doi:10.1002/ece3.6586)
Supplement: Supplementary file 1 — Supplementary Material [file ECE3-10-9192-s001.docx]

**Electronic supplementary material**

**Appendix**

***Non-trivial equilibrium***

By setting the right-hand side of Eqs. 1a-f in the main text to zero, the nontrivial equilibrium is obtained. In C-limited system, the equilibrium is:

$N^{*}=\frac{a_{M}D^{*}M^{*}\left\{\delta-e\left(q-1+\delta\right)\right\}+\left(1-e\right)\delta\left(a_{Cb}M^{*}C_{b}^{*}+a_{Cg}P^{*}C_{g}^{*}\right)+I}{rP^{*}+l_{N}},$ (S-1a)

$P^{*}=\frac{l_{Cg}+m_{Cg}}{ea_{Cg}},$ (S-1b)

$C_{g}^{*}=\frac{rN^{*}-\left(l_{P}+m_{P}\right)}{a_{Cg}},$ (S-1c)

$D^{*}=\frac{\left(1-\delta\right)\left(1-e\right)a_{Cb}M^{*}C_{b}^{*}+\left(1-\delta\right)\left(1-e\right)a_{Cg}P^{*}C_{g}^{*}+m_{P}P^{*}+m_{M}M^{*}+m_{Cg}C_{g}^{*}+m_{Cb}C_{b}^{*}}{\left\{e\left(1-\delta\right)+\delta\right\}a_{M}M^{*}+l_{D}},$ (S-1d)

$M^{*}=\frac{l_{Cb}+m_{Cb}}{ea_{Cb}},$ (S-1e)

$C_{b}^{*}=\frac{qea_{M}D^{*}-\left(l_{M}+m_{M}\right)}{a_{Cb}},$ (S-1f)

The equilibrium of N-limited system is same as (S-1a-f) except for$N^{*}=\frac{\left(1-e\right)\delta\left(a_{M}D^{*}M^{*}+a_{Cb}M^{*}C_{b}^{*}+a_{Cg}P^{*}C_{g}^{*}\right)+I}{rP^{*}+r_{M}M^{*}+l_{N}}$ (S-2a)

and

$C_{b}^{*}=\frac{ea_{M}D^{*}+r_{M}N^{*}-\left(l_{M}+m_{M}\right)}{a_{Cb}}.$ (S-2b)

The equilibrium in C-limited system is feasible if and only if:

$I>\frac{e\left(q-1\right)a_{M}D^{*}M^{*}}{\left(1-e\right)\delta\left(a_{M}D^{*}M^{*}+a_{Cb}M^{*}C_{b}^{*}+a_{Cg}P^{*}C_{g}^{*}\right)}$, (S-3a)

*N**> (*l_P_ + m_P_*)/*r*, (S-3b)

and

*D** > (*l_M_ + m_M_*)/*qea_M_* (S-3c)

This suggests that nutrient input rate, the nutrient pool size, and detritus pool size must be larger than the threshold.

The equilibrium in N-limited system is feasible if and only if:

*N**> (*l_P_ + m_P_*)/*r*, (S-4a)

and

*N** > (*l_M_ + m_M_ − ea_M_D**)/*r_M_* (S-4b)

This suggests that nutrient input rate is not key for the feasibility and nutrient pool size is a critical for the feasibility.

***Comparison of equilibrium with or without decomposers***

Here, I compare the equilibrium with or without decomposers. To do this, the consumers of decomposers are not considered (*C_b_* = 0). The model without decomposers is the followings:

$\frac{dN}{dt}=I-l_{N}N-rNP+a_{M}D,$ (S-5a)$\frac{dP}{dt}=rNP-\left(l_{P}+m_{P}\right)P-a_{Cg}PC_{g},$ (S-5b)$\frac{dC_{g}}{dt}=e_{Cg}a_{Cg}PC_{g}-\left(l_{Cg}+m_{Cg}\right)C_{g},$ (S-5c)$\frac{dD}{dt}=m_{P}P+m_{Cg}C_{g}-a_{M}D-l_{D}D,$ (S-5d)

The definitions of parameters are same with those of Eqs. (1) in the text.

I focus on the equilibrium of detritus and decomposers because it is a key difference in the two systems with or without decomposers. First, consider the simplest case without consumers of producers (*C_g_* = 0). In this case, the equilibrium of detritus in the system (S-5) is

$D^{*}=\frac{m_{P}\left\{rI-l_{N}\left(l_{P}+m_{P}\right)\right\}}{r\left\{l_{D}m_{P}+l_{P}\left(l_{D}+a_{M}\right)\right\}},$ (S-6)

The equilibrium of detritus and decomposers in the system (Eqs. 1 in the text) is

*C-limited case*

$D^{*}=\frac{l_{M}+m_{M}}{a_{M}eq},$ (S-7a)

$M^{*}=\frac{A_{1}\left(l_{P}+m_{P}\right)-eqrm_{P}I}{rA_{2}},$ (S-7b)

where

$$A_{1}=\frac{rl_{D}\left(l_{M}+m_{M}\right)}{a_{M}}+eql_{N}m_{P},$$

$$A_{2}=\left\{-\delta+e\left(-1+q+\delta\right)\right\}l_{P}m_{M}+l_{M}\left[\left\{-e+\left(-1+e\right)\delta\right\}l_{P}-eqm_{P}\right].$$

*N-limited case*

$D^{*}=\frac{r\left(l_{M}+m_{M}\right)-r_{M}\left(l_{P}+m_{P}\right)}{a_{M}er},$ (S-7c)

$M^{*}=\frac{A_{3}\left(l_{P}+m_{P}\right)-erm_{P}I}{A_{4}+A_{5}},$ (S-7d)

where

$$A_{3}=el_{N}m_{P}+l_{D}\left(r\left(l_{M}+m_{M}\right)-r_{M}\left(l_{P}+m_{P}\right)\right)/a_{M},$$

$$A_{4}=rl_{M}\left[\left\{-e+\left(-1+e\right)\delta\right\}l_{P}-em_{P}\right],$$

$$A_{5}=l_{P}\left[\left(-1+e\right)r\delta m_{M}-\left\{-e+\left(-1+e\right)\delta\right\}r_{M}\left(l_{P}+m_{P}\right)\right].$$

Here, consider a limit where the decomposition rate *a_M_* is very large. In such case, it is clear that *D^*^* in all models (S-6, S-7a, S-7c) with or without decomposers becomes zero. However, it is clear that *M^*^* (S-7b, S-7d) can converge to a positive constant (not shown because of the complexity).

Second, consider the case with consumers of producers (*C_g_* > 0). In this case, the equilibrium of detritus in the system (S-5) is

$D^{*}=\frac{C_{g}^{*}\left(-1+e\right)P^{*}\left(-1+\delta\right)a_{Cg}+C_{g}^{*}m_{Cg}+P^{*}m_{P}}{a_{M}+l_{D}},$ (S-8)

*C-limited case*

$D^{*}=\frac{l_{M}+m_{M}}{a_{M}eq},$ (S-9a)

$M^{*}=\frac{-C_{g}^{*}P^{*}\left(1-e\right)\left(1-\delta\right)a_{g}+D^{*}l_{D}-C_{g}^{*}m_{Cg}-P^{*}m_{P}}{\left(l_{M}+m_{M}\right)\left\{e\left(-1+\delta\right)-\delta\right\}/eq+m_{M}},$ (S-9b)

*N-limited case*

$D^{*}=\frac{l_{M}+m_{M}-r_{M}N^{*}}{a_{M}e},$ (S-9c)

$M^{*}=\frac{-C_{g}^{*}P^{*}\left(1-e\right)\left(1-\delta\right)a_{g}+D^{*}l_{D}-C_{g}^{*}m_{Cg}-P^{*}m_{P}}{a_{M}D^{*}\left(e\left(-1+\delta\right)-\delta\right)+m_{M}}$ (S-9d)

Here, consider a limit where the decomposition rate *a_M_* is very large. In such case, it is clear that *D^*^* in (S-9a) becomes zero. Although it is nontrivial, $\lim_{a_{M}\to\infty} D^{*}=0$ is also shown in (S-8, S-9c) if we use a limit function in Wolfram Mathematica. If we use this software system, we can show *M^*^* in (S-9b, S-9d) can be a positive constant (not shown because of the complexity).

Even in the full model (Eqs. 1 in the text), $\lim_{a_{M}\to\infty} D^{*}=0$ is shown by a limit function in Wolfram Mathematica. On the other hand, it is clear that $\lim_{a_{M}\to\infty} M^{*}$is a positive constant because *M^*^* is not a function of *a_M_* (see S-1e).

**Supplemental figures (Fig. S1-10)**

**Fig. S1**. Relationships between total nutrient input and resilience in C-limited system. (a-c) *a_Cg_* = 1.5. (d-f) *a_Cg_* = 2. In (a, d), (b, e), and (c, f), *l* = 0.01, 0.1, and 0.2, respectively. Other information is same as Fig. 2 a-c.

**Fig. S2**. Relationships between total nutrient input and resilience in N-limited system. (a-c) *a_Cg_* = 1.5. (d-f) *a_Cg_* = 2. In (a, d), (b, e), and (c, f), *l* = 0.02, 0.1, and 0.2, respectively. Other information is same as Fig. 2 d-f.

**Fig. S3**. Relationships between decomposition rate and resilience in the systems with or without consumers of decomposers. (a-c) C-limited. (d-f) N-limited. Red and blue lines indicate systems with or without the consumers of decomposers, respectively. In the grey regions, coexistence does not occur. In (a, d), (b, e), and (c, f), *l* = 0.01, 0.1, and 0.5, respectively. Parameter values are *I* = 2, *r =* 2, *e =* 0.25, *δ* = 0.5, *q =* 1.2, *a_Cg_ =* 1, *a_Cb_ =* 1, *r_M_ =* 0.1, *m_P_* = 0.1, *m_Cg_* = 0.1, *m_M_* = 0.1, *m_Cb_* = 0.1, *l_P_* = 0.1, *l_Cg_* = 0.1, *l_M_* = 0.1, and *l_Cb_* = 0.1.

**Fig. S4**. Relationships between decomposition rate and resilience in the full model. (a-c) C-limited. (d-f) N-limited. Colors indicate different values of *a_Cb_*. *l =* 0.5. Other parameter values are same as Fig. S3.

**Fig. S5**. Relationships between the decomposition rate (*a_M_*) and resilience with varying ecosystem openness in C-limited system. (a-f) are the effects of parameters, *I*, *r*, *m_P_*, *m_Cg_*, *m_M_*, and *m_Cb_*, respectively. Other information is same as Fig. 2a.

**Fig. S6**. Relationships between the decomposition rate (*a_M_*) and resilience with varying ecosystem openness in C-limited system. (a-h) are the effects of parameters, *l_P_*, *l_Cg_*, *l_M_*, *l_Cb_*, *a_Cg_*, *a_Cb_*, *q*, and *δ*, respectively. Other information is same as Fig. 2a.

**Fig. S7**. Relationships between the decomposition rate (*a_M_*) and resilience with varying ecosystem openness in N-limited system. (a-h) are the effects of parameters, *I*, *r*, *m_P_*, *m_Cg_*, *m_M_*, *m_Cb_*, *l_P_*, and *l_Cg_*, respectively. Other information is same as Fig. 2b.

**Fig. S8**. Relationships between the decomposition rate (*a_M_*) and resilience with varying ecosystem openness in N-limited system. (a-g) are the effects of parameters, *l_M_*, *l_Cb_*, *a_Cg_*, *a_Cb_*, *r_M_*, *q*, and *δ*, respectively. Other information is same as Fig. 2b.

**Fig. S9**. Relationships between the decomposition rate (*a_M_*) and resilience with varying ecosystem openness in donor-controlled systems. (a) C-limited. (b) N-limited. In donor-controlled systems, decomposition function is obtained by removing *M* from terms describing decomposer consumption. Other information is same as Fig. 2.

**Fig. S10**. Relationships between decomposition rate and resilience in the system with second consumers. (a) C-limited. (b) N-limited. The new variables of *C_g_*_2_ and *C_b_*_2_, which represent population size of second consumers in green and brown worlds, respectively. The new terms, *δ*(1 − *e*){*a_Cg_*_2_*C_g_C_g_*_2_+ *a_Cb_*_2_*C_b_C_b_*_2_}, −*a_Cg_*_2_*C_g_C_g_*_2_, (1 − *δ*)(1 − *e*)(*a_Cg_*_2_*C_g_C_g_*_2_+ *a_Cb_*_2_*C_b_C_b_*_2_) + *m_Cg_*_2_*C_g_*_2_ + *m_Cb_*_2_*C_b_*_2_, −*a_Cb_*_2_*C_b_C_b_*_2_, are added into Eq. 1a, 1c, 1d, and 1f, respectively. The new added differential equations are: d*C_g_*_2_/dt = *ea_Cg_*_2_*C_g_C_g_*_2_ − (*m_Cg_*_2_ *+ l_Cg_*_2_)*C_g_*_2_ and d*C_b_*_2_/dt = *ea_Cb_*_2_*C_b_C_b_*_2_ − (*m_Cb_*_2_ *+ l_Cb_*_2_)*C_b_*_2_, where each parameters are defined as those of second consumers. Colors indicate different values of *l*. Parameter values are: *I* = 2, *r =* 2, *e =* 0.25, *q =* 1.2, *a_Cg_ =* 1, *a_Cg_*_2_ *=* 1, *a_Cb_ =* 1, *a_Cb_*_2_ *=* 1.2, *r_M_ =* 1, *m_P_* = 0.1, *m_Cg_* = 0.1, *m_M_* = 0.1, *m_Cb_* = 0.1, *l_P_* = 0.1, *l_Cg_* = 0.1, *l_M_* = 0.1, and *l_Cb_* = 0.1. In C- and N-limited systems, *δ =* 0.5 and *δ =* 0.2 are assumed.
